# Towards Green Computing Oriented Security: A Lightweight Postquantum Signature for IoE

**DOI:** 10.3390/s21051883

**Published:** 2021-03-08

**Authors:** Rinki Rani, Sushil Kumar, Omprakash Kaiwartya, Ahmad M. Khasawneh, Jaime Lloret, Mahmoud Ahmad Al-Khasawneh, Marwan Mahmoud, Alaa Abdulsalm Alarood

**Affiliations:** 1 School of Computer and Systems Sciences, Jawaharlal Nehru University (JNU), New Delhi 110067, India; rinki32_scs@jnu.ac.in (R.R.); skdohare@mail.jnu.ac.in (S.K.); 2 Department of Computer Science, Clifton Campus, Nottingham Trent University, Nottingham NG11 8NS, UK; 3 Department of Mobile Computing, Amman Arab University, Amman 11953, Jordan; a.khasawneh@aau.edu.jo; 4 Integrated Management Coastal Research Institute, Universitat Politecnica de Valencia, 46022 Valencia, Spain; jlloret@dcom.upv.es; 5 School of Computing and Digital Technologies, Staffordshire University, Stoke ST4 2DE, UK; 6 Faculty of Computer & Information Technology, Al-Madinah International University, Kuala Lumpur 57100, Malaysia; mahmoud@outlook.my; 7 Department of Computer and Information Technology, Faculty of Applied Studies, King Abdulaziz University, Jeddah 21589, Saudi Arabia; mmamahmoud@kau.edu.sa; 8 College of Computer Science and Engineering, University of Jeddah, Jeddah 21959, Saudi Arabia; aasoleman@uj.edu.sa

**Keywords:** energy efficiency, green computing, lightweight security, Internet of Things

## Abstract

Postquantum cryptography for elevating security against attacks by quantum computers in the Internet of Everything (IoE) is still in its infancy. Most postquantum based cryptosystems have longer keys and signature sizes and require more computations that span several orders of magnitude in energy consumption and computation time, hence the sizes of the keys and signature are considered as another aspect of security by green design. To address these issues, the security solutions should migrate to the advanced and potent methods for protection against quantum attacks and offer energy efficient and faster cryptocomputations. In this context, a novel security framework Lightweight Postquantum ID-based Signature (LPQS) for secure communication in the IoE environment is presented. The proposed LPQS framework incorporates a supersingular isogeny curve to present a digital signature with small key sizes which is quantum-resistant. To reduce the size of the keys, compressed curves are used and the validation of the signature depends on the commutative property of the curves. The unforgeability of LPQS under an adaptively chosen message attack is proved. Security analysis and the experimental validation of LPQS are performed under a realistic software simulation environment to assess its lightweight performance considering embedded nodes. It is evident that the size of keys and the signature of LPQS is smaller than that of existing signature-based postquantum security techniques for IoE. It is robust in the postquantum environment and efficient in terms of energy and computations.

## 1. Introduction

The Internet of Everything (IoE) is an interconnection of smart devices, business processes and data structures without any human intervention [1]. It expands applications from digital sensor tools to smart and self-configuring intelligent nodes in distributed hardware to enrich the lives of people [2]. In such smart networks, information security is of paramount importance as all the decisions and actions depend on the accuracy and credibility of the received data [3]. The public key infrastructure (PKI) plays a critical role in information security. In PKI, however, both the sender and the receiver authenticate each other with the help of certificates obtained from the certificate authority. This process can be time-consuming and complex. Identity-based cryptography (IBC) schemes remove these barriers and use public strings such as email addresses or domain names for data encryption and signature verification, instead of digital certificates [4]. The security of IBC depends on solving some mathematical problems such as integer factorization and discrete logarithms. Major recent signature schemes depend on these two mathematical problems, which are infeasible to solve on any classical computer. However, these problems can easily be solved by quantum computers in polynomial time. For instance, Shor’s quantum algorithm can solve the integer factorization in polynomial time [5]. Moreover, it can not only forge a signature but also recover private keys. Thus, such system poses serious threats to the modern cryptography. To effectively block these threads, many cryptographers are developing new quantum-resistant algorithms that are unbreakable in the era of quantum computers. Several postquantum cryptography (PQC) classes have been proposed which are currently believed to be quantum resistant, namely: lattice-based [6,7,8], hash-based [9], code-based PQC [10] and isogeny-based [11].

Over the past few years, isogeny-based cryptography has been gaining a lot of momentum owing to its small key sizes. Various isogeny-based cryptosystems have been published for public key encryption and key exchange protocols [12,13] but later have been broken by a subexponential quantum attack. Recently, a key exchange scheme based on supersingular isogeny Diffie–Hellman (SIDH) has been proposed, for which there is no known subexponential quantum attack [14] and is much faster than ordinary isogeny. SIDH uses supersingular elliptic curves for key exchange and public key encryption [15,16]. Isogeny-based cryptosystems have also been used for digital signatures such as the strong designated verifier signature [17] and the undeniable signature [18]. However, the feasibility of these schemes on resource-constrained devices is not known. The compressed digital signature scheme reduces the public and private key sizes to 336 and 48 bytes, respectively, for the 128-bit quantum security level. Unfortunately, these primary signature schemes are slower than other quantum signature techniques due to their larger signature sizes.

The prime issues in security by green computing for IoE applications are related to the key size, signature and the encryption computation of the postquantum based cryptosystems, which must be kept compact to reduce energy consumption and computation time [19]. Most postquantum based cryptosystems require higher order of magnitude longer keys to provide current the level of protection, which are substantial enough to impact energy requirements and computation time [20]. The use of isogeny curve based postquantum cryptography is considered to be the most practicable solution to the energy required for the shortest key’s computation. To efficiently exploit the resistant capability of postquantum cryptography, we use a supersingular isogeny curve and ID-based signature for postquantum cryptography, which requires much shorter keys to maintain the same level of protection and provides user friendly access to the system. In addition to this, it can also reduce the overall energy and time needed for the crypto operations in comparison to postquantum based cryptosystems and therefore facilitate appropriate replacement in sensors, handheld devices, and IoE applications.

In this context, a lightweight postquantum ID-based signature (LPQS) scheme using a supersingular isogeny curve for secure data transmission in the IoE environment is presented. The design of the LPQS scheme aims to provide a signature scheme for the postquantum cryptography and to reduce the complexity of the system with the consumption of fewer system resources. The LPQS scheme uses the identity of the client for the initialization of the process. Further, this scheme uses two isogeny curves for verification to provide double-fold secure encryption. The main contributions of the scheme can be summarized as:

- Firstly, a system model for post quantum security is presented considering its applicability in IoE environments.
- Secondly, the four phases of the execution of the proposed framework LPQS are detailed, where compressed curves are used to reduce the size of keys and the validation of the signature depends on the commutative property of curves.
- Thirdly, the unforgeability of LPQS under an adaptively chosen message attack is proved and security analysis is performed to show its resistance against various cyberattacks.
- Finally, performance analysis and experimental validation of the proposed framework are performed under software simulation environment to assess its lightweight performance in realistic IoE environments considering the embedded nodes.

The rest of the paper is organized as follows. Section 2 presents the recent review of nonquantum and postquantum cryptographic techniques. Section 3 presents the details of the proposed lightweight security framework LPQS. In Section 4 discusses security analysis and experimental comparative performance evaluation considering range of metrics, followed by conclusions presented in Section 5.

## 2. Related Work 

For security in sensor networks, Jao et al. [14] proposed a cryptosystem based on supersingular isogenies for encryption and key exchange which is much faster in contrast to the ordinary isogenies based schemes. This work was further extended by Plut et al. [15] and gave a public key exchange scheme which includes zero-knowledge proof of identity. This model achieves approximately 0.06 s per key exchange runtime operation as presented in test scenario. Costela et al. [16] proposed more efficient algorithms for computing isogenies. This algorithm have claimed to run 2.9 times faster than the scheme by Plut et al. Earlier, the isogeny based cryptographic functions were available only for key exchange protocol or public key encryption scheme. Thereafter, Galbarith et al. [17] proposed the first signature scheme based on supersingular isogeny problems. This scheme is resistant to chosen message attacks in the random oracle model. To achieve a small signature size a time–space trade-off is used which deteriorates the performance of the scheme. Hence, to improve the performance, a signature scheme based on isogeny-based zero-knowledge proof have been suggested which further reduces signature size with small key sizes [18,19]. However, this scheme suffers from poor performance compared to the other postquantum schemes. 

Elliptic Curve Cryptosystem (ECC) based models have been very prominent in IoT. Considering the efficiency of ECC, Malasri et al. [20] gave an authentication scheme for medical sensor networks. As a result, this model could maintain confidentiality and message integrity. In this key management scheme, every step computes the message authentication code, which depletes the resources and delays the packets’ processing at the receiver end. Further, Oliveira et al. [21] gave a secure scheme for sensor networks based on IBC and proved it to be practical for resource-constrained nodes. In this scheme, senders broadcast their identities with no security measure and it allows adversaries to broadcast several fake identities and helps them to launch denial-of-service (DoS) attacks. This attack reduces the power of low computation devices. Tan et al. [22] proposed an identity-based cryptography scheme for the security of body sensor networks. This approach uses a hash function for public key generation and stores the key on the sensor’s flash memory. Further, this model uses the public key for the computation of elliptic curve encryption/decryption using the Elliptic Curve Digital Signature Algorithm (ECDSA). For public key computation, this scheme requires more storage, energy and computation time. Sankaran et al. [23] gave an IDKEYMAN which uses IBC for wireless body area networks parties to exchange symmetric keys. The pairwise symmetric keys support the minimization of energy consumption.

In addition, this approach provides security from replay attacks by using ephemeral values. This technique does not provide protection against other attacks like selective forwarding, Sybil, etc. Li et al. [24] proposed a biometric-based scheme where physiology signals like electrocardiogram are used to create keys and transmits them in a safe mode. This biometric-based scheme improves the network security and increases the lifetime of the model by using fuzzy commitment and an arbitrated-based approach. However, this approach is limited to a wireless body area network only. Ma et al. [25] proposed a practical access control technique based on IBC for the Internet of Things (IoT). This signcryption scheme provides a reduction in energy and less computation cost with large area applicability [26]. 

Public key cryptographic algorithms depend on the hardness of integer factorization and discrete log problems. However, these algorithms will be vulnerable to attacks from quantum computers. Considerable research has been conducted for postquantum cryptography. Among various postquantum techniques, the lattice-based signatures [27] scheme is prominent and based on the hardness of NTRU (Nth degree Truncated polynomial Ring Units) problems with no algebraic structure. The limitation of these techniques is that they have large public and private keys and are not feasible for many practical applications. Another candidate for postquantum cryptography is multivariate-based signatures [28]. These signatures are based on the multivariate quadratic polynomial problem. These models have a smaller signature but large key sizes and are difficult to scale to higher security levels [29]. Furthermore, hash-based techniques have small key sizes but are inefficient in terms of speed. Hence, none of the abovementioned techniques are feasible for the IoE environment [30]. Because of the small key size, isogeny-based cryptography is a suitable candidate for the IoE environment. An isogeny-based cryptosystem depends on the difficulty of computing isogeny between two given curves of the same order. 

The first isogeny-based cryptosystem for public key encryption and the key exchange was a traditional model without considering quantum computing. However, Childs et al. [31] proposed a postquantum algorithm that computes ordinary isogenies in subexponential time. Since the algorithm relies on the commutative property of endomorphism rings, it does not apply to the supersingular singular case [32]. Feo et al. [33] gave a signature model using class group actions for the 128-bit security level. This model uses only a 1 KB signature size and maintains adequate security in the random oracle model. Parrilla et al. [34] have suggested a unified coprocessor framework in order to run the ECC on IoT devices. The group key support strategy is also incorporated for reducing the communication overhead in key distribution. Similarly, to deal with malfunctioning of the IoT enabled systems, Hussein et al. [35] investigated a secure protocol to maintain the secrecy rate in IoT environments and to reduce the energy consumption at IoT nodes. However, both these ECC frameworks are vulnerable against quantum attacks as edge centric faster and efficient security enabler nodes have not been considered to support the security operations of resources constrained IoT nodes. Quantum centric security analyses have been also missing in the analytical investigation of these approaches. 

## 3. Lightweight Postquantum Signature Scheme for IoE

### 3.1. Preliminaries—Basics of Supersingular Iosgency Curve

Initially, we briefly introduce the supersingular isogeny curve that has been used to design the proposed signature scheme and its problems to prove its resistance against cyberattacks. We consider two elliptic curves $E_{A},\;E_{B}$ over a finite field $\;F_{q}$ also used in [36,37]. An isogeny φ: $E_{A}\to E_{B}$ is a nonconstant morphism that preserves the group structure [38]. The degree of an isogeny φ is equal to the degree of φ as a morphism. An isogeny of degree ℓ is called a ℓ-isogeny [39,40]. If φ is separable, then deg φ = #ker φ. If isogeny is separable between two curves, we say that they are isogenous [41]. Tate’s theorem [42,43] is that two curves $E_{A},E_{B}$ over $F_{q}$ are isogenous if and only if $\;\#E_{A}(F_{q})=\#E_{B}(F_{q})$. An isogeny can be identified by its kernel in such a way that for every finite subgroup G of $\;E_{A}$, there is a unique $E_{B}$ and a separable isogeny φ: $E_{A}\to E_{B}$ with kernel G such that φ: $E_{B}≅E_{A}/G$. To obtain subgroup G we can use Vélu’s formulae. 

Isogenies with the same domain and range are called as endomorphisms. The set of endomorphisms is maximal order either to quaternion algebra or to an imaginary quadratic field. The curve is supersingular for the first case; otherwise, the curve is ordinary. In the case of a supersingular elliptic curve, there is always a curve in the isomorphism class defined over $F_{p^{2}}$, thus its j-invariant is over $F_{p^{2}}$. One can construct a so-called isogeny graph for any prime  ℓ ≠ p, where an edge and vertex are associated with an l-isogeny and j-invariant, respectively. Next, we present a few hard problems related to supersingular elliptic curves over $F_{p^{2}}$.

Problem 1 (computational supersingular isogeny ($CSSI_{A}$) problem): suppose $\Phi_{A}:\;E_{0}\to E_{A}$ to be an isogeny with kernel ($P_{A}+\left[\alpha \right]Q_{A}$) where α chose at random from ᴢ/$l_{A}^{e_{A}}z$ and not divisible by $l_{A}$. Find a generator $G_{A}$ of ($P_{A}+\left[\alpha \right]Q_{A}$) where $\left\{\;E_{A},\;\Phi_{A}\left(P_{C}\right),\;\Phi_{A}\left(Q_{C}\right)\right\}$ is given.

Problem 2 (computational supersingular isogeny ($CSSI_{C}$) problem): suppose $\Phi_{C}:\;E_{0}\to E_{C}$ to be an isogeny with kernel ($P_{C}+\left[\beta \right]Q_{C}$) where β chose at random from ᴢ/$l_{C}^{e_{C}}z$ and not divisible by $l_{C}$. Find a generator $G_{C}$ of ($P_{C}+\left[\beta \right]Q_{C}$) where $\left\{\;E_{C},\;\Phi_{C}\left(P_{A}\right),\;\Phi_{C}\left(Q_{A}\right)\right\}$ is given.

Problem 3 (supersingular isogeny Diffie–Hellman (SIDH) problem): let $\Phi_{A}:\;E_{0}\to E_{A}$ be an isogeny with kernel $\langle P_{A}+\left[\alpha \right]Q_{A}\rangle ,$ and $\Phi_{C}:\;E_{0}\to E_{C}$ be an isogeny with kernel $\;\langle P_{C}+\left[\beta \right]Q_{C}\rangle$, where  α,β are chosen at random from ᴢ/$l_{A}^{e_{A}}z$ and ᴢ/$l_{C}^{e_{C}}z$, respectively. {$E_{A},\;\Phi_{A}\left(P_{C}\right),\;\Phi_{A}\left(Q_{C}\right)$,$E_{C},\;\Phi_{C}\left(P_{A}\right),\;\Phi_{C}\left(Q_{A}\right)$} be given, find j-invariant of $E_{0}/$〈$P_{A}+\left[\alpha \right]Q_{A},\;P_{C}+\left[\beta \right]Q_{C}$)〉.

Problem 4 (supersingular isogeny auxiliary point ccomputation ($SIAPC_{A}$)): suppose $\Phi_{A}:\;E_{0}\to E_{A}$ to be an isogeny with kernel ($P_{A}+\left[\alpha \right]Q_{A}$) where α chose at random from ᴢ/$l_{A}^{e_{A}}z$ and is not divisible by $l_{A}$. The supersingular isogeny auxiliary point computation problem is to find the auxiliary point $\Phi_{A}\left(P_{C}\right)$ and $\Phi_{A}\left(Q_{C}\right)$, where $\;\{E,E_{A},\;P_{A},Q_{A},P_{C},Q_{C}$} are given.

Problem 5 (supersingular isogeny auxiliary point computation ($SIAPC_{C}$)): suppose $\Phi_{C}:\;E_{0}\to E_{C}$ to be an isogeny with kernel ($P_{C}+\left[\beta \right]Q_{C}$) where β is chosen at random from ᴢ/$l_{C}^{e_{C}}z$ and is not divisible by $l_{C}$. The supersingular isogeny auxiliary point computation problem is to find the auxiliary point $\Phi_{c}\left(P_{A}\right)$ and $\Phi_{C}\left(Q_{A}\right)$, where {$E,E_{A},\;P_{A},Q_{A},P_{C},Q_{C}$} are given.

A signature scheme consists of three polynomial time algorithms: key generation, registration, and validation. We prove the security of the scheme using the existential unforgeable under an adaptively chosen message attack (EU-ACMA) (32). A forger and a challenger play a game where the forger uses the public key and signing oracle model. The forger issues signature queries to the sign oracle to generate a signature $\sigma_{i}$ of message $m_{i}$ and the oracle sends $\sigma_{i}$ to the forger. The attack is considered successful when the forger produces a valid signature and message pair different from those generated from the query oracle.

**Definition** **1.** *A digital signature scheme is existentially unforgeable under an adaptively chosen message attack (EU-ACMA) if any adversary Ã cannot produce a valid message–signature pair in polynomial time with access to the signing oracle.*

Setup: Suppose we have a function KeyGen to output key pair (*pk*, *sk*), and challenger give the *pk* to the adversary Ã.

Queries: The adversary Ã issues signature queries to sign oracle Ś to generate valid signature $\underset{.}{\sigma_{1}},\ldots ,\;\sigma_{i}$ corresponding to messages $M_{1},\ldots ,M_{i}$.

Output: Finally, adversary Ã generates a valid message signature pair ($M^{*},\;\sigma^{*}$) and wins the game if $M^{*}\notin \;M_{i}$. 

The signature scheme is secure if probability to distinguish between simulated signature and real signature is negligible for adversary Ã with access to signing oracle $\left(Sign_{sk}\left(.\right)\right)$ i.e.,

$$\mathrm{Pr}\;\left[\begin{array}{c} \left(pk,\;sk\right)\leftarrow keyGen\left(1^{n}\right)\\ \left(M_{i},\sigma_{i}\right)\leftarrow {Ã}^{Sign_{sk}\left(.\right)}\left(\mathrm{pk}\right)\;\\ Verify_{PK}\left(M,\sigma \right)=1\;and\;M^{*}\;\notin \;M_{i} \end{array}\right]\;\leq negl\;\left(\lambda \right)$$

### 3.2. System Model

**Figure 1:**
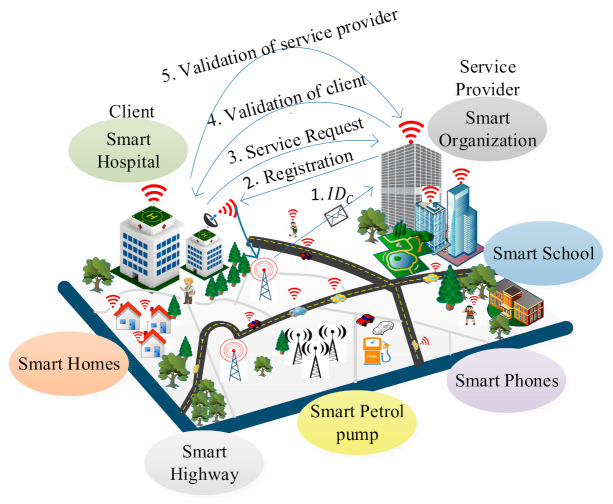
A system model for the lightweight postquantum ID-based signature (LPQS) framework.

We consider an IoE environment in which several heterogeneous smart nodes such as an individual human, an organization, sensors, vehicles, smart watches, smart phones are deployed as shown in Figure 1. We classify these smart nodes into two main categories: service provider and client. In the IoE environment, a client can be an organization, an individual human or any device that wants to access services such as health reports collection, banking, e-commerce. The client encrypts the data with its signature and sends it to the service provider. The service provider allows authentic clients to access the service. A service provider provides an organization with three servers: the key generation server, the database server, and the validation server. For individual clients, the key generation server generates the global parameters and public–private keys. The database server maintains the data and the validation server helps in authenticating the clients. The service provider generates appropriate rights using a tag machine and performs key generation, encryption/decryption using the supersingular isogeny curves. It issues the rights to clients based on the service such as a client can view only his/her data for a particular period. The Internet of Everything (IoE) is considered as superset of Internet of Things (IoT). IoE covers the wider concept of connectivity where network intelligence at the edge devices makes it a more complex network tha then IoT. So, basically, it can be considered as an extension of the IoT in terms of network management and network intelligence.

To ensure secure data transmission between a service provider and clients, and to reduce the complexity of the system with less consumption of the system resources, we present a LPQS scheme for secure data transmission for an IoE environment. The scheme uses supersingular isogeny curves for the postquantum cryptography signature. The proposed scheme consists of four phases: initialization, registration, signature, and validation. In the first phase, the service provider initializes all the parameters for global access. In the second phase, the service provider calculates the basis points for the clients using the ID of an individual client. The client performs the signature on the data with the help of the service provider in the signature phase. In the validation phase, the clients and service providers validate each other using the two isogeny curves. We want to clarify that “green” means a reduction in the computing requirement for providing security in the IoE environment. The proposed framework LPQS reduces the size of keys and signature for enabling security in the IoE. It also uses keys which can be used for longer period and are flexible in use, further reducing computation at the IoE nodes. Thus, green design means it is energy-efficient for the IoE nodes, as well as computing power efficient for the coordinator nodes at the edge.

### 3.3. Lightweight Post Quantum Signature

Firstly, in the initialization phase, the service provider initializes the system by setting all the global parameters as a set $\left\{p,\;E,\;P_{A},\;Q_{A},\;I_{A}\left(2\right),\;I_{B}\left(3\right),\;n,\;m\right\}$, where the description and use of every parameter is given in Table 1. Isogeny-based cryptosystem uses supersingular elliptic curves over characteristic p, where p is a prime of the form $\;2^{n}\times 3^{m}\times f\;\pm 1$. Here, n, m are positive integers such that $2^{n}≃\;3^{m}$ and f is a small cofactor to ensure p as a prime. This special form of p allows us to efficiently compute isogenies, as given in the next sections. The global parameters generated by service provider include $\left\{p,\;E,\;P_{A},\;Q_{A},\;I_{A}\left(2\right),\;I_{B}\left(3\right),\;n,\;m\right\}$ over the curve E of finite field $F_{p^{2}}$ of characteristics p with $p^{2}$ element. The service provider selects a random integer α, such that $0\;\leq \;\alpha \;\leq 2^{n}$. The random number α is kept secret as the service provider’s secret key. The service provider uses an ephemeral secret key, which changes in every session to support nontraceability. Fix points $P_{A},\;Q_{A}\epsilon E\left[2^{n}\right]$ such that group $\langle P_{A},\;Q_{A}\rangle$ generated by $P_{A}$ and $Q_{A}$ in the whole group $E\left[2^{n}\right]$. The elliptic curve points $\left(P_{A},\;Q_{A}\right)$ are the global parameters of the supersingular isogeny-based cryptosystem. $GT_{A}\;=P_{A}+\left[\alpha \right]Q_{A}$, where $\langle GT_{A}\rangle$ is the generator of a kernel of service provider which creates a secret subgroup of *E*[$2^{n}$]. $E_{A}=E/\langle GT_{A}\rangle$ is the elliptic curve that is the image curve under the isogeny {$\Phi_{A}$}.

**Figure 2:**
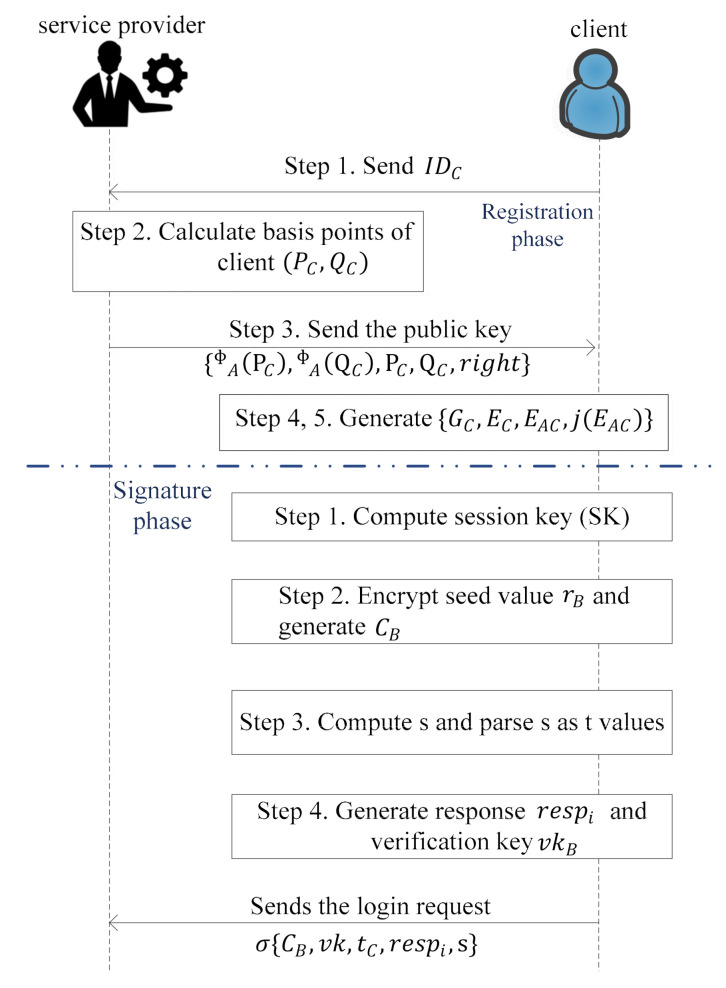
Flow diagram of registration and signature.

Secondly, the in registration phase, service provider performs the registration with the help of the client (C) to provide access to the facility/services of the service provider in the IoE environment as shown in Figure 2 and the steps are:

Step 1. The client sends its identity $ID_{C}$ generated randomly to the service provider through a public channel.
Step 2. After receiving the $ID_{C}$, the service provider calculates basis points of client i.e.,$\;Q_{C}$ and $P_{C}$ using the $ID_{C}$ and right, which are assigned by service provider as expressed by Equations (1) and (2).
  (1) $$Q_{C}=H\left(ID_{C}\;||\;f\right)$$
  (2) $$P_{C}=H\left(right\;||\;ID_{C}\right)⊕p$$
  where, H is a fixed hash function, and rights are the authority assigned to the client. The notation ⊕ is the xor function, and || is a concatenation operation.
Step 3. The service provider generates the public key of client as $\left\{\Phi_{A}\left(P_{C}\right),\;\Phi_{A}\left(Q_{C}\right),\;P_{C},\;Q_{C},\;right\right\}$ and sends it to the client.
Step 4. Upon receiving $\left\{\Phi_{A}\left(P_{C}\right),\;\Phi_{A}\left(Q_{C}\right),\;P_{C},\;Q_{C},right\right\}$, the client selects a random number as a secret key from $0\;\leq \;\beta \;\leq \;3^{m}$. The generator $G_{C}$ for the kernel of the client is expressed as given by Equation (3).
  (3) $$G_{C}=P_{C}+[\beta ]Q_{C}$$
  where $P_{C}$ and $Q_{C}$ are the basis for $E_{C}$ and $E_{C}=E/\langle G_{C}\rangle$.
Step 5. The client computes the image curve $E_{AC}$ and also computes the shared secret value $j\left(E_{AC}\right)$, where $j\left(E_{AC}\right)$ is the j-invariant of the image curve $E_{AC}$.

Thirdly, in the signature phase, the client does the following four steps to sign message m which is shown in Figure 2.

**Figure 3:**
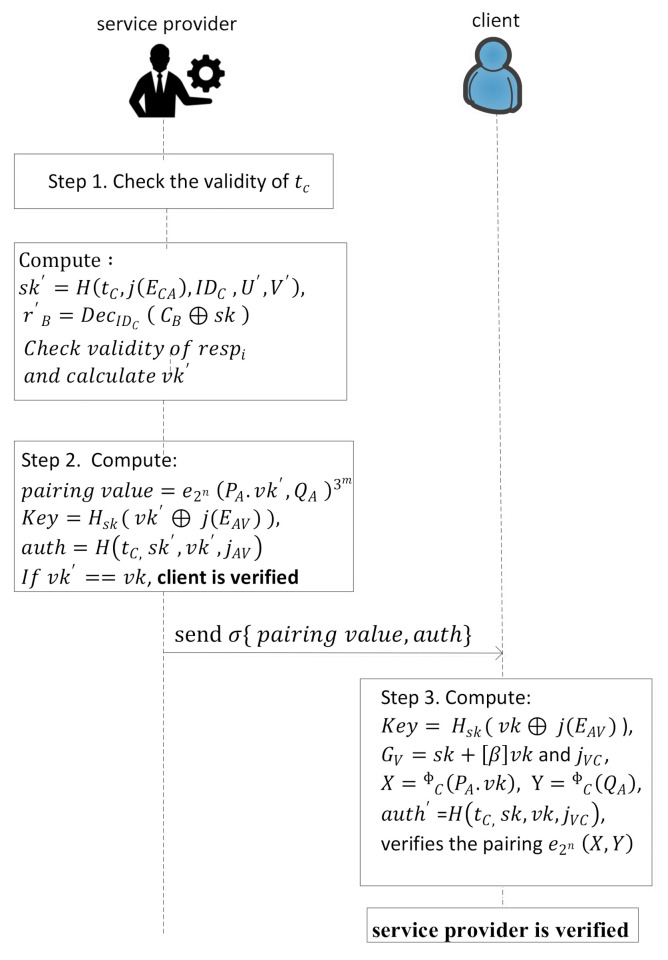
The work flow of client and service provider validation.

Step 1. The client calculates the $sessionkey\;\left(sk\right)=H\left(t_{C},j\left(E_{AC}\right),ID_{C},\;U,\;V\right)$, where $U=\Phi_{C}\left(P_{A}\right),\;V=\Phi_{C}\left(Q_{A}\right)$ and $t_{C}$ is the timestamp.
Step 2. Further, encrypt the seed value $r_{B}$ as expressed by Equation (4).
  (4) $$C_{B}=Enc_{ID_{C}}\left(r_{B}\;⊕sk\right),\;\mathrm{for}\;1\;\leq \;B\;\leq \;t$$
Step 3. Compute $s=\;H\left(m,\;C_{1},\ldots ,C_{t}\right)$. Parse s as t values $CH_{B}\in {\left\{0,1\right\}}^{C}$.
Step 4. If $CH_{i}=1$ then response $resp_{i}=\left(G_{C},\Phi_{A}\left(G_{C}\right)\right)$ else $resp_{i}=\left(\Phi_{c}\left(G_{A}\right)\right)$. $\Phi_{A}\left(G_{C}\right)$ is only calculated by the service provider and verification key $\left(vk_{B}\right)=h(t_{C},\;j\left(E_{AC}\right),\;ID_{C},\;r_{B},CH_{B,}s)$ for 1≤B≤t. The client sends the login request $\sigma \{C_{B},\;vk,t_{C},resp_{i},s\}$ to the service provider.
  In this last validation phase the service provider and the client validate each other, which is shown in Figure 3 with stepwise description as follows.

Step 1. The service provider checks the validity of $t_{C}$ of received signature σ and if it is valid then proceeds further; otherwise the service provider rejects the request. After checking the $t_{C}$ validity, the service provider calculates the image of the client with the help of its basis as, $\Phi_{A}\left(P_{C}\right)=\Phi_{C}\left(P_{A}\right)=U^{\prime }$, $\Phi_{A}\left(Q_{C}\right)=\Phi_{C}\left(Q_{A}\right)=V^{\prime }$ and also computes $sk^{\prime }=H\left(t_{C},\;j\left(E_{CA}\right),ID_{C},U^{\prime },V^{\prime }\right)$ and ${r^{\prime }}_{B}$ as expressed by Equation (5).
  (5) $${r^{\prime }}_{B}=Dec_{ID_{C}}\left(C_{B}⊕sk\right)$$
  for *i* = 1 to *t*, parse *s* as *t* values and check if $CH_{i}=1,$ then parse $resp_{i}$. Check if $resp_{i}$ has order $3^{m}$ and if $G_{C}$ generates $E_{C}$ and $\Phi_{A}\left(G_{C}\right)$ generates $E_{CA}$. If $CH_{i}=0,$ then check if $resp_{i}$ has order $2^{n}$ and generates $E_{AC}$ and $vk^{\prime }=h\left(t_{C},\;j\left(E_{CA}\right),{r^{\prime }}_{B},\;ID_{C},\;CH_{B,}s\;\right).$ If $vk^{\prime }$ is equal to vk then clientC is authenticated.
Step 2. The service provider computes $pairingvalue=e_{2^{n}}{\left(P_{A}.vk^{\prime },\;Q_{A}\right)}^{3^{m}}$ and develops the key and authentication using sk and vk as expressed in Equations (6) and (7), and computes the value of $\Phi_{A}\left(sk^{\prime }\right),\;\Phi_{A}\left(pk^{\prime }\right),\;E_{V}$ and $j_{AV}$ (as shown in Figure 3) and send $\sigma \left\{\;pairingvalue,\;auth\right\}$ to the client.
  (6) $$Key=H_{sk}\left(vk^{\prime }⊕\;j(E_{AV}\right)),$$
  (7) $$auth=H\left(t_{C,\;}sk^{\prime },\;vk^{\prime },\;j_{AV}\right)$$
Step 3. After receiving the signature, the client verifies the authenticity of the service provider and computes $Key=\;H_{sk}\left(vk⊕\;j(E_{AV}\right)$) and $G_{V}=sk+\left[\beta \right]vk$ and $j_{VC}$ as shown in Figure 3. Further, it calculates $X=\Phi_{C}\left(P_{A}.vk\right),\;Y=\Phi_{C}\left(Q_{A}\right)$,$auth^{\prime }$ =$H\left(t_{C,\;}sk,\;vk,\;j_{VC}\right)$ and also verifies the pairing $e_{2^{n}}\left(X,Y\right).$ Now the service provider is also verified.

## 4. Security Analysis and Experimental Results

### 4.1. Mathematical Security Analysis

**Theorem** **1.** *The digital signature LPQS is EU-ACMA in the quantum random oracle model with constraint relation expressed in Equation (8).*

(8) $$\varepsilon \;(1/2^{n})\;\left(1-\left(q_{q}/2^{k}-4q_{h}-q_{s}\right)\right)\left(1-q_{q}/2^{\left|F_{P^{2}}\right|}\right)\leq Pr\left[C\right]$$

*where*

$$1/2^{n}<\frac{1}{4},q_{q}/2^{\left|F_{P^{2}}\right|}<\frac{1}{3},\;\mathrm{so}\;\frac{\varepsilon }{2}\left(1-\left(q_{q}/2^{k}-4q_{h}-q_{s}\right)\right)\leq Pr\left[C\right].$$

**Proof.**  Suppose an adversary A exists in the system who can produce valid LPQS signatures. It takes system parameters {$\;p,\;E,\;P_{A},\;Q_{A},I_{A}\left(2\right),\;I_{B}\left(3\right),\;n,\;m,\;P_{c},\;Q_{c}$}, public keys $(E_{A},\Phi_{A}\left(P_{C}\right),\;\Phi_{A}\left(Q_{C}\right)$) and a verifier $\left(E_{B},\Phi_{C}\left(P_{A}\right),\;\Phi_{C}\left(Q_{A}\right)\right)$. The adversary make queries q to the oracle of client C with queries of a signing oracle (Ȿ), and a verifying oracle (ν), and a hashing oracle (ℋ). The adversary A aims at producing $\sigma \{C_{B},\;vk,t_{C},resp_{i},s\}$ for $M^{*}\notin \;M_{i}$. To generate a regular LPQS signature, he first calculates the basis point U, V. Then he computes sk and encrypts the seed value. Let $CH_{0},\;CH_{1}$ represent the possible outcome of the challenge ch=0,1, respectively, with the cardinality of c. If ch=0, then $resp=\left(\Phi_{c}\left(G_{A}\right)\right)$ otherwise $resp=\left(G_{C},\Phi_{A}\left(G_{C}\right)\right)$. The verifier will accept the signature if the resp contains the right order. □

We now calculate the success probability of adversary A. The probability of the secret value of the signing oracle $\left(0\leq \;\alpha \leq 2^{n}\right)$ is guessed successfully is 1/$2^{n}.$ The probability adversary A can produce a valid signature by inquiring $q_{q}$ queries to the signing oracle are $\left(1-\left(q_{q}/2^{k}-4q_{h}-q_{s}\right)\right)$ where $q_{h},q_{s}$ denotes the total number of queries for a hashing and signing oracle and k is the output length of the hash function h. The $4q_{h}$ queries are required to calculate $sk^{\prime },vk^{\prime },key,$ and auth. Another probability that A solves the SSCDH problem is at least $\left(1-q_{q}/2^{\left|F_{P^{2}}\right|}\right)$. Therefore, the successful simulation of A happens with a probability constraint relation as expressed in Equation (8). This contradicts with the hardness of the SIDH problem (Poblem 3). Thus, there is no adversary A that could forge a signature under an adaptively chosen message attack.

### 4.2. Theoretical Security Analysis

In this subsection, we present theoretical analysis of the LPQS scheme to prove its resistance against various cyberattacks and it is described as:

(1) Mutual authentication: the client and the service provider share the messages $\;\{C_{B},\;vk,t_{C}$} and $\left\{\;pairingvalue,\;auth\;\right\}$, respectively. vk depends on the $j\left(E_{AC}\right)$ which is a SIDH problem (Problem 3) and it is hard to find the value of $j\left(E_{AC}\right)$. Furthermore, $C_{B}$ is also difficult for the adversary to obtain as it contains sk. Similarly, auth cannot be calculated because of the hardness of SIDH. Therefore, our scheme provides mutual authentication.
(2) Anonymity: in the proposed scheme, the client’s identity is hidden in the message $\left\{C_{B},\;vk,t_{C}\right\}$, where $vk=h(t_{C},\;j\left(E_{AC}\right),\;ID_{C},\;r_{B}),C_{B}=Enc_{ID_{C}}\left(r_{B}\;⊕sk\right)$, $sk=H\left(t_{C},j\left(E_{AC}\right),ID_{C},\;U,\;V\right).$ To find the value of the client’s identity, the adversary has to calculate the $j\left(E_{AC}\right)$ which is a SIDH problem (Problem 3). Therefore, our scheme is secure to maintain the anonymity of the client.
(3) Nontraceability: suppose the adversary stores the value of $\left\{C_{B},\;vk,t_{C}\right\}$ and the $\left\{\;pairingvalue,\;auth\;\right\}$ exchange between client and service provider. As α and β are the ephemeral keys and changing in each session separately, even if the adversary guesses the private key it will not be possible to find the auxiliary point {$\Phi_{c}\left(P_{A}\right)$,$\Phi_{C}\left(Q_{A}\right),\;\Phi_{A}\left(P_{C}\right)$,$\Phi_{A}\left(Q_{C}\right)$} as given in Problem (4),(5).
(4) No verification table: in the proposed scheme, no verification table has been maintained for the mutual authentication between the client and the service provider.
(5) Session key agreement: the client and the service provider both generate the session key, $key=h\;\left(sk,\;vk,\;j(E_{AC}\right))$, where $sk=H\left(t_{C},j\left(E_{AC}\right),ID_{C},\;U,\;V\right),vk=h(t_{C},\;j\left(E_{AC}\right),\;ID_{C},\;r_{B}),\;U=\Phi_{A}\left(P_{C}\right)$, $V=\Phi_{A}\left(Q_{C}\right)$. For an adversary it is not possible to create a valid login session because of the Problem (4) and (5). So, our scheme could provide the session key agreement.
(6) Perfect forward secrecy: perfect forward secrecy is provided by $j(E_{AC})$ and is explained in Theorem 1.
(7) Attack resistance: we present that our scheme is resistant to impersonation attacks, replay attacks, modification attacks, stolen verifier attacks and the man-in-the-middle attacks.
  (a) Impersonation attack: according to Theorem 1, we can claim that any adversary without any secret key cannot generate a generator as described in problem (1), (2) and without the generator no auxiliary point can be calculated as described in problem (4) and (5). So, only a valid client and service provider can create a login message or response $\{C_{B},\;vk,t_{C}\},\;\left\{\;pairingvalue,\;auth\right\}$. Then the client and the service provider can check the validity of each other by checking the $\left\{\;pairingvalue,\;auth\right\}$, and $\left\{C_{B},\;vk,t_{C}\right\}$ and can find out if any adversary is present in the system.
  (b) Replay attack: in the LPQS scheme, the client access the service by generating the message $\;\{C_{B},\;vk,t_{C}\}$. After receiving the message, the service provider checks the freshness of $t_{C}$, before executing the other steps. If in any case adversary generates $t_{C}$ and captures the packet $\left\{C_{B},\;vk,t_{C}\right\}$, the adversary would not be able to calculate the key without knowing the private key of the client i.e., β. Furthermore, an adversary cannot use the same login message in another session as clients and service providers use a different key {α, β} in each session. So, the client and service provider could find the replay attack by checking the {pairingvalue,auth} and $\{C_{B},\;vk,t_{C}\}.$
  (c) Modification attack: the service provider can detect the modification attack by checking the validity of the signature $\left\{C_{B},\;vk,t_{C}\right\}$. Similarly, the clients can check the validity of $\left\{pairingvalues,auth\right\}$.
  (d) Stolen verifier table attack: no table is maintained in our scheme by the client or the service provider. So, no such attack is possible.
  (e) Man-in-middle attack: due to the mutual authentication, no man-in–the middle attack is possible.
(8) Due to the usage of supersingular isogeny curves, we can effectively compress the keys and signature size. The infinite field $F_{p^{2}}$ elements used to transmit the points $\Phi_{A}\left(P_{C}\right),\;\Phi_{A}\left(Q_{C}\right)$ are rather large compared to the size of the integer coefficients. However, we have used compressed curves which can be represented by one field element. The key basis calculated by the nodes need not be published as a public parameter, as long as all nodes are able to generate the same basis independently by a predefined algorithm. It also supports perfect forward-secrecy, nontraceability and anonymity as detailed in Section 4.2. In summary, to efficiently exploit the resistant capability of postquantum cryptography, we have used a supersingular isogeny curve and an ID-based signature for postquantum cryptography that requires much shorter keys to maintain the same level of protection and provides user friendly access to the security system.

### 4.3. Computation Cost Analysis

The computation cost of the LPQS scheme is given in detail for the public key, the private key and the signature. In this computation, we have neglected the lightweight operations like XOR and string concatenation, as we know primes p have the form of $\;2^{n}.3^{m}.f\;\pm 1,$ such that $2^{n}≃\;3^{m}$. We compute the cost in terms of λ bits for the λ bits of a quantum computer. We assume p has 6λ bits length. All values are calculated for 128-bit security. Our scheme uses Montgomery curves $E:By^{2}=x^{3}+Ax^{2}+x$, where A–coefficient is sufficient for isogeny computation. The isomorphism classes of the Montgomery form have the same Kummer line. So, both can be represented by one field element, requiring 12 λ-bits. We compare LPQS in the terms of the sizes of public and private keys, and signatures with variants of lattice, multivariate and isogeny, and is shown in Table 2.

(1) Public Keys

In LPQS, public keys contain $\left\{\Phi_{A}\left(P_{C}\right),\;\Phi_{A}\left(Q_{C}\right),\;P_{C},\;Q_{C},\;right\right\}$, where $P_{C}\;and\;Q_{C},\;$ are the points on the elliptic curve E of order $3^{m}$ calculated by the service provider using XOR and concatenation operations. So, its cost is negligible and right needs no operation. Further, torsion basis ($\Phi_{A}\left(P_{C}\right),\;\Phi_{A}\left(Q_{C}\right))$ requires three 3 λ-bits coefficients and 12 λ-bits for the curve. Thus, the public key requires 21 λ-bits. For 128-bit quantum, it needs 336 bytes (21 × 128 = 2688 bits). Other postquantum techniques such as lattice-based (6) and multivariate (28) need 11,653 bytes and 417,408 bytes, respectively.

(2) Private keys

Private keys contain the two generators $GT_{A,\;},\;G_{Av,\;}$ as described in the Section 4. The private key $GT_{A}\left(GT_{A}=P_{A}+\left[\alpha \right]Q_{A}\right)$ can be represented as a single coefficient α with respect to the basis point $P_{A},\;Q_{A}$ and it requires 3 λ-bits. So, for two generators we need 6 λ-bits and for 128-bit security level we need 96 bytes (6 × 128=768 bits).

(3) Signature

The signature of the client includes $\{C_{B},\;vk,t_{C}\},$ where $C_{B}$ is an encrypted representation of the random seed value $r_{B}$ and $(sk=H\left(t_{C},j\left(E_{AC}\right),ID_{C},\;U,\;V\right)$). As we discussed in the previous section, the computation cost of U, V is 6 λ-bits and the hash function is 3 λ-bits. The J-invariant ($j\left(E_{AC}\right)$) requires 6 λ-bits to store the value in the 128-bit computer. Further, $vk\;\left(vk=h(t_{C},\;j\left(E_{AC}\right),\;ID_{C},\;r_{B}\right))$ takes 3 λ-bits for the hash function. So, the total cost will be 18 λ-bits. The service provider’s signature includes the $\left\{\;pairingvalue,\;auth\right\},$ where the mapping cost is negligible and auth =$\;H_{key}\left(t_{C,\;}sk^{\prime },\;vk^{\prime },\;j_{AV}\right)$. The hash function requires 3λ-bits and similarly the $sk^{\prime },\;vk^{\prime }$ need 15 and 3λ-bits, respectively, and Key = $H_{sk}\left(vk^{\prime }⊕\;j(E_{AV}\right)$) requires 3 λ-bits. Thus, the total signature cost of the client and service provider is 39 λ-bits. Thus, on average, our scheme requires 21λ-bits (336 bytes) for a public key, 6 λ-bits (96 bytes) for private key and 39 $\lambda^{2}$-bits (39 × 128 × 128 = 79,872 bits) which is equal to 9984 bytes for a signature to achieve 128-bit of quantum security. Comparatively, the signature size is larger than the public and private key because for the signature we use two torsion groups ($E_{A},\;E_{C}$) to increase the hardness of the isogeny problem, but it requires more storage space.

### 4.4. Experimental Implementation and Discussion

In this section, we evaluate the performance of the ID-based LPQS scheme for secure data transmission in the IoE environment. The C implementation done in (36) is further extended to include the signature scheme introduced in this paper. For the comparison analysis, we compute the energy consumption, computation time, and CPU cycles taken by the key generation, signing, and verification. We use the C language in the Microsoft Visual Studio 2013 platform on Intel(R) Core(TM) i7-8700 CPU @3.20 GHZ with ×64-based processor, running Windows 10 to implement the proposed scheme. Intel Power Gadget 3.7.0 is used to measure the execution time and energy consumption of LPQS. We also used Raspberry Pi-based IoE nodes to measure the performance of the embedded devices. Our scheme uses Montgomery curves $E:By^{2}=x^{3}+Ax^{2}+x$, where the A–coefficient is sufficient for isogeny computation. The comparative analysis is performed with state-of-the-art nonquantum and postquantum techniques.

#### 4.4.1. Nonquantum Schemes

**Figure 4:**
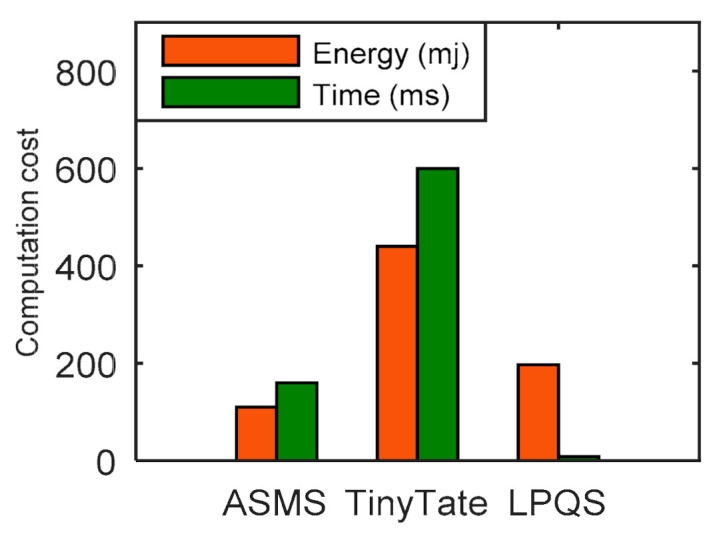
Computation cost of nonqunatum techniques for energy and time consumption.

In this subsection, we compare the energy and time of LPQS with predicate nonquantum signature schemes ASMS (20) and TinyTate (21) for 128-bit nonquantum security level. Nonquantum security 128-bit is approximately equal to 85-bit security level. ASMS and TinyTate use the elliptic curve $y^{2}=x^{3}+x$. We have considered one ID and one byte of data transmission using AES-128. In terms of energy, ASMS and TinyTate take 110 mJ and 440 mJ, respectively, to perform key generation, signature and verification, while LPQS needs 196.85 mJ to perform the same task, which is 123% more efficient than TinyTate. The total time consumption of LPQS is 8.057 ms. ASMS and TinyTate take 2410 ms and 600 ms, respectively, as is shown in Figure 4. So, LPQS is approximately 300 and 74 times faster than ASMS and TinyTate, respectively. The reason for less computation time is the use of the isogeny curve. It takes less time to perform addition, subtraction and multiplication and hence the overall time reduces effectively. It is noted that 128-bit nonquantum security can be achieved at 85-bit quantum security level with a reasonable tradeoff between energy and time.

#### 4.4.2. Postquantum Schemes

In this section, we evaluate the performance of the LPQS scheme with state-of-the-art schemes. The performance of the LPQS scheme is evaluated in terms of time for key generation, signature and verification, which are iterated 10 times for prime p503, p751, p1019, and p1533. A comparative analysis of the energy with nonisogeny signature schemes SPHINCS (9) and Rainbow (30) are presented. The total number of clock cycles is also analysed and compared with the isogeny based schemes Efficient Algorithms for Supersingular Isogeny (EASI) (16), Microsoft’s Supersingular Isogeny Diffie-Hellman (MSIDH) (36), Efficient Post-Quantum Undeniable signature (EPQU) (39), and Key Compression for Isogeny-Based cryptosystems (KCIB) (40). In LPQS, we use supersingular elliptic curves with prime $p=\;2^{n}.3^{m}.f\;\pm 1$. For prime p503, n is 250, m is 159, f is 1 and it provides 83 bit quantum security, which is approximately equal to 85-bit quantum security, and other prime values are shown in Table 3.

**Figure 5:**
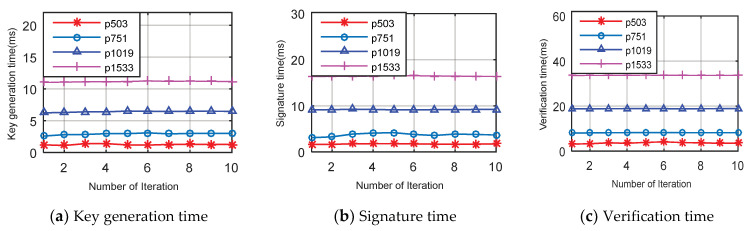
Various computation time of different phase vs. number of iterations with different *p* values.

The computation time of key generation for different p values is shown in Figure 5a and Table 4. All results are run for 10 iterations. For p503, p751, p1019, and p1533 the key generations’ average running times are 1.25, 2.96, 6.45 and 11.17 ms, respectively. Further, the average running times of signature generation for p503, p751, p1019, and p1533 are 1.75, 3.9, 9.20 and 16.44 ms, respectively. Signature time is more than key generation time because we use two isogeny curves (i.e., $\Phi_{A},\Phi_{C})$ and only one isogeny is used for key generation (i.e., $\Phi_{A}$). In Figure 5c, the computation time of verification is shown and it is clear that average running times for p503, p751, p1019 and p1533 are 3.45, 8.17, 18.84 and 33.66 ms, respectively. Verification needs three times more computation time than key generation and two times more computation time than the signature phase. Thus, most of the computation time is spent on verification because the signature size is larger than the public and private keys and in addition, two isogeny operations and one pairing operation are also performed.

**Figure 6:**
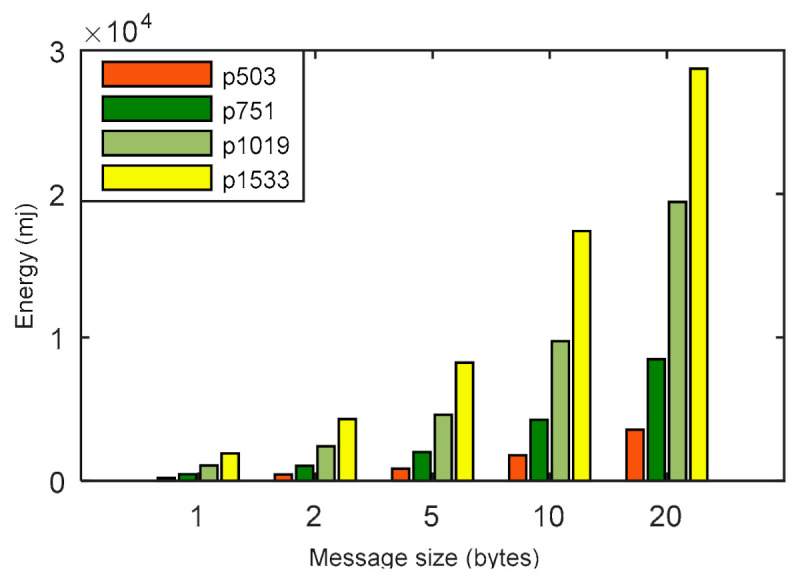
Comparison for energy (in millijoules) with message sizes (in bytes) for various p sizes.

**Figure 7:**
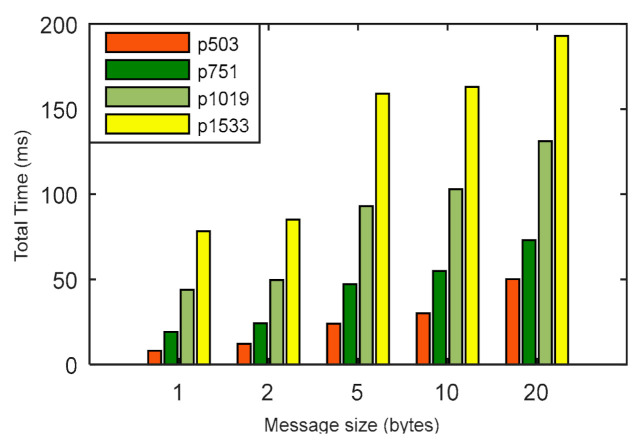
Total time to perform the operations considering different message sizes (in bytes) for various p sizes.

In Figure 6, the energy consumption of the LPQS is shown for different message sizes. The message size’s impact on the energy consumption and is clear from Figure 6 and Table 5. For a 5 byte message, the maximum and minimum energy consumptions are 848.440 mJ and 8243.409 mJ, respectively. Energy consumption is increasing exponentially with the increase of the message size and security level. Hence, for a security level of 256-bits and a message size of 20 bytes, the energy consumption is 34,733.251 mJ. The total times taken to complete the processes for p1019 are 43.82, 49.64, 93.00, 103.00 and 131.21 ms for 1, 2, 5, 10 and 20 bytes of message, respectively. It is clear from Figure 7 and Table 6 that the total time is increasing linearly with increase in the size of the messages.

**Figure 8:**
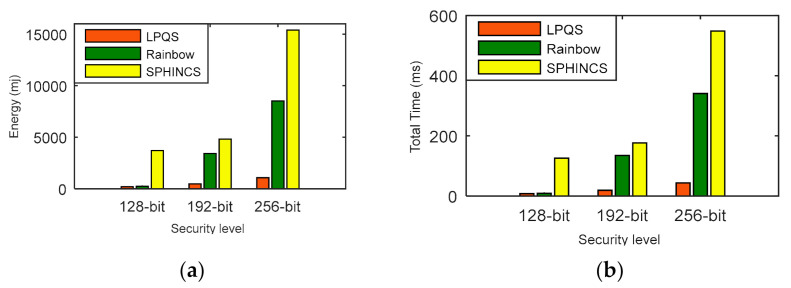
(**a**) Energy consumption, (**b**) computation time comparison of LPQS with nonisogeny based methods.

We have compared the energy consumption and time computation of LPQS with the nonisogeny signature scheme for 128-bit, 192-bit and 256-bit security levels. In this comparison, we are considering message size as one byte for one ID. For 128-bit security level, Rainbow and SPHINCS need energy of 234.76 mJ and 3706.66 mJ, respectively. LPQS consumes 196.854 mJ, which is approximately 1.1 times and 19 times more efficient than Rainbow and SPHINCS, respectively, and is shown in Figure 8a and Table 7. For 256-bit security level, LPQS needs 1070.64 mJ while Rainbow and SPHINCS take 8518.95 mJ and 15,394.60 mJ, respectively. Further time taken by Rainbow and SPHINCS for 128-bit security are 9.12 ms and 125.9 ms, respectively. For the same security level LPQS needs 8.057 ms, which is approximately 15 times faster than SPHINCS.

**Figure 9:**
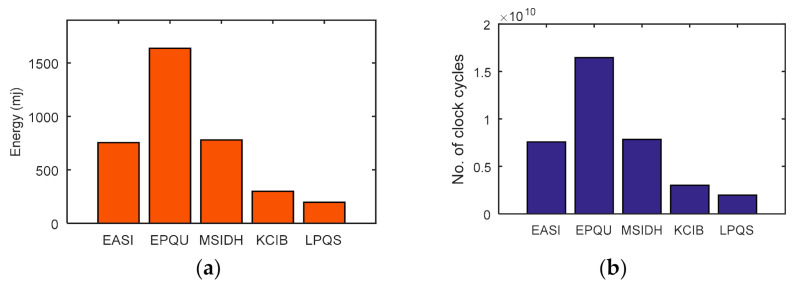
(**a**) Energy consumption; (**b**) clock cycle comparison with isogeny based postquantum schemes.

For 256-bit security level, Rainbow and SPHINCS take 340.86 ms and 548.30 ms. However, LPQS needs 43.821 ms for 256-bit security level, as is shown in Figure 8b. These values may be different for different processors. However, LPQS has smaller public and private key sizes (as shown in Table 2), and it consumes less energy and time, and is clear from Figure 8. As shown in Figure 9, EASI takes 754.102 mJ of energy and 7580 million CPU cycles for SIDH key exchange, while EPQU needs energy of 1637.039 mJ and 16,455 million cycles for an undeniable signature. MSIDH and EASI consume 7836 and 3009 million cycles, respectively, for the complete process, while LPQS takes 1976 million cycles and needs 196.854 mJ of energy for the signature, which is the least among the state-of-the-art schemes. The reason for the lower amount of energy and fewer CPU cycles is the usage of two isogeny curves instead of one, which takes the previously computed values for the second verification.

**Figure 10:**
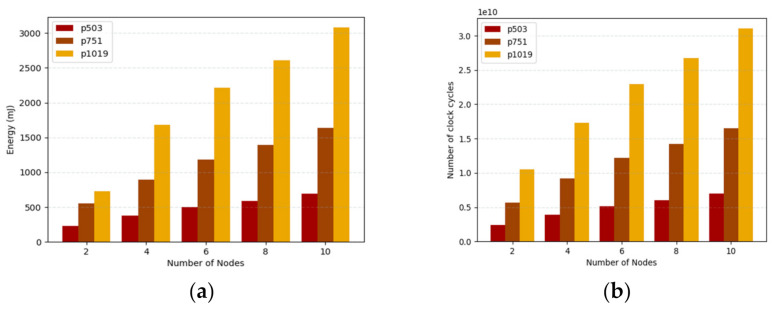
(**a**)Energy consumption, (**b**) number of clock cycles in million cycles with number of nodes.

The energy consumption of the embedded devices implemented in Raspberry Pi for different numbers of nodes is shown in Figure 10a. In this environment, the numbers of clients are increasing from 2 to 10. For two clients the energy consumption is 233.109 mJ and for six clients 497.805 mJ for p503. Further, the energy consumption for p1019 with eight clients is 2612.706 mJ. As we know, the keys are computed once and used for a long period of time. For the signature, the clients need only one pairing and hash operation, which takes less energy for computation. Figure 10b shows the number of clock cycles consumed for a number of nodes ranging from 2 to 10. For p751, the number of clock cycles taken are 1391 and 1640 million cycles for 8 and 10 nodes, respectively. The LPQS consumes fewer CPU cycles because it uses previously computed isogeny values for the next computation.

## 5. Conclusions and Future Work

In this paper, we presented a lightweight postquantum ID-based signature scheme using the supersingular elliptic curve isogeny for the IoE environments. We use the ID for the calculation of the basis for clients and two isogenies for the verification of service provider and clients. Compressed curves are used to reduce the size of keys and validation of signature depends on the commutative property of curves. In comparison with the nonquantum schemes, LPQS outperforms state-of-the-art techniques in terms of time, CPU cycle and energy. Further, Montgomery curves reduced the public and private keys, and signature sizes. We performed a thorough analysis of postquantum schemes on X86-64 system and Raspberry Pi enabled embedded nodes. The results have clearly shown that the LPQS is feasible for embedded devices. Finally, in comparison with the state-of-the-art techniques, the LPQS scheme is more efficient and secure. In the future, we will extend our scheme to investigate how to represent the elliptic curves efficiently and use the three-party id-based signature scheme based on the supersingular isogeny curve for future networks such data or content focused networking [44] and vehicular communication [45].

## Figures and Tables

**Table 1:** Nomenclature.

Symbol	Description
p	Prime number
E	Elliptic curve over finite field F
$P_{A},\;Q_{A}\;$	Elliptic curve basis points
$\Phi_{A},\;\Phi_{C}\;$	Isogeny for supersingular curve $E_{A},\;E_{C}$
n, m	Positive integers such that $2^{n}≃\;3^{m}$
$GT_{A}$	Generator of a kernel of service provider
f	Small cofactor to ensure p as a prime
$r_{B}$	Seed value
${ID}_{C}$	Identity of client
||	Concatenation operator
⊕	Xor operator

**Table 2:** Postquantum signatures scheme comparison in bytes with 128-bit quantum security.

Scheme	Public Key Size	Private Key Size	Signature Size
Lattice-based (6)	11,653	6769	2444
Lattice-based (33)	7168	2048	5120
Multivariate-based (28)	417,408	14,208	48
Multivariate-based (29)	81,800	8900	337
Multivariate-based (30)	136,100	101,300	79
Hash-based (9)	1000	1000	41,000
Isogeny-based (11)	768	48	141,312
LPQS	336	96	9984

**Table 3:** Public parameters with comparative nonquantum and quantum security (bits).

$p=2^{n}.3^{m}.f\;\pm \;1$	NonQuantum Security (bit)	Quantum Security (Bit)
$p503=2^{250}3^{159}-1$	125	83
$p751=2^{372}3^{239}-1$	186	124
$p1019=2^{508}3^{319}.35-1$	253	168
$p1533=2^{776}3^{477}-1$	378	252

**Table 4:** Computation time of different phases for different prime values.

**Key Generation Time with Number of Iterations**										
**P**	**1**	**2**	**3**	**4**	**5**	**6**	**7**	**8**	**9**	**10**
*p*503	1.20	1.11	1.39	1.40	1.20	1.18	1.27	1.29	1.27	1.27
*p*751	2.60	2.81	2.85	2.97	2.96	3.10	2.95	2.99	3.02	3.01
*p*1019	6.25	6.30	6.39	6.40	6.53	6.49	6.45	6.49	6.51	6.45
*p*1533	11.02	11.07	11.12	11.13	11.17	11.25	11.20	11.21	11.19	11.17
**Signature Time with Number of Iterations**										
*p*503	1.65	1.69	1.77	1.79	1.81	1.75	1.73	1.69	1.71	1.76
*p*751	3.10	3.30	3.90	4.10	4.20	3.80	3.60	3.90	3.80	3.70
*p*1019	9.14	9.20	9.25	9.21	9.15	9.13	9.17	9.19	9.22	9.21
*p*1533	16.35	16.39	16.41	16.44	16.51	16.52	16.49	16.44	16.39	16.38
**Verification Time with Number of Iterations**										
*p*503	3.10	3.30	3.70	3.50	3.90	4.10	3.80	3.70	3.50	3.60
*p*751	8.05	8.11	8.17	8.21	8.23	8.20	8.19	8.16	8.17	8.20
*p*1019	18.76	18.81	18.83	18.86	18.89	18.84	18.81	18.83	18.85	18.86
*p*1533	33.58	33.62	33.65	33.69	33.70	33.68	33.64	33.63	33.63	33.64

**Table 5:** Message size vs. energy consumption (mJ) for different p values.

Message Size (Bytes)	1	2	5	10	20
P503	196.854	442.921	848.440	1791.371	3574.868
P751	467.154	1051.096	2013.433	4251.101	8483.516
P1019	1070.640	2408.940	4614.458	9742.824	19,442.822
P1533	1912.624	4303.404	8243.409	17,404.878	34,733.251

**Table 6:** Message size vs. time (ms) for different p values.

Message Size (Bytes)	1	2	5	10	20
P503	8.05	12.16	24.0	30.14	50.14
P751	19.12	24.19	47.10	54.90	73.01
P1019	43.82	49.64	93.00	103.00	131.21
P1533	78.29	85.12	159.00	163.00	192.98

**Table 7:** Comparison of total energy (mJ) with postquantum techniques at different security level.

Security Level	Energy (mJ)			Total Time (ms)		
	128-bit	192-bit	256-bit	128-bit	192-bit	256-bit
Rainbow	234.76	3421.63	8518.95	9.12	134.93	340.86
SPHINCS	3706.66	4812.19	15,394.60	125.90	176.57	548.30
LPQS	196.58	467.15	1070.64	8.057	19.12	43.82

## Data Availability

The experimental data and associated settings will be made available to researchers and practitioners on individual request to corresponding author, with the restrictions that it will solely be used for further research in literature progress. As the associated research data is being further utilized for development research by the team.
